# Recurrent Pleural Solitary Fibrous Tumor: A Case Report and Brief Literature Review

**DOI:** 10.3390/reports9030229

**Published:** 2026-07-17

**Authors:** Alexandra Paraschiv, Teodora-Oana Iordache, Elena-Doina Magheran, Vasile Grigorie, Daniel Dumitru Dinescu, Ioana-Mădălina Moșteanu, Ana-Luiza Iorga

**Affiliations:** 1 Department of Pulmonology, “Marius Nasta” Institute of Pulmonology, 050159 Bucharest, Romania; teodora-oana.iordache0525@rez.umfcd.ro (T.-O.I.); madalina.mosteanu@yahoo.com (I.-M.M.); analuiza_iorga@yahoo.com (A.-L.I.); 2 Faculty of Medicine, “Carol Davila” University of Medicine and Pharmacy, 050474 Bucharest, Romania; 3 Department of Anatomical Pathology, “Marius Nasta” Institute of Pulmonology, 050159 Bucharest, Romania; elena.magheran@yahoo.com; 4 Department of Thoracic Surgery, “Marius Nasta” Institute of Pulmonology, 050159 Bucharest, Romania; vasile.grigorie@marius-nasta.ro (V.G.); dinescudumitrudaniel@yahoo.com (D.D.D.); 5 Doctoral School, University of Medicine and Pharmacy of Craiova, 200349 Craiova, Romania

**Keywords:** solitary fibrous tumor, pleural neoplasms, tumor recurrence, STAT6, Demicco score, risk stratification

## Abstract

**Background and Clinical Significance:** Solitary fibrous tumors (SFTs) of the pleura are rare mesenchymal neoplasms accounting for less than 5% of pleuro-pulmonary tumors. Recurrence after complete resection of benign SFT is exceptional, with an estimated rate of approximately 3%. **Case Presentation:** We describe a 78-year-old woman who developed a late recurrence 10 years after resection of a giant benign pleural SFT. The new pleura-based mass was confirmed as SFT by CT-guided biopsy demonstrating spindle-cell morphology, patternless architecture, staghorn vasculature, and STAT6/CD34 positivity, consistent with intermediate-risk disease by Demicco scoring. Surgical tumorectomy was successfully performed. **Conclusions:** This case illustrates the unpredictable long-term behavior of pleural SFTs and reinforces the importance of extended imaging surveillance even in initially benign lesions. An updated review of contemporary classification systems, molecular mechanisms, and management strategies is included.

## 1. Introduction and Clinical Significance

Solitary fibrous tumors (SFTs) represent less than 5% of all pleuro-pulmonary neoplasms and are encountered predominantly in elderly individuals, most frequently in the sixth and seventh decades of life [1].

No association has been established with occupational exposure to ionizing radiation, asbestos, or other toxic agents, and no predisposing risk factors have been identified to date. In contrast to the majority of pulmonary neoplasms, whose pathogenesis is closely related to tobacco smoke and electronic cigarette aerosol exposure, SFTs appear to develop independently of smoking behavior and environmental respiratory factors, further underscoring their distinct mesenchymal nature and unique pathogenetic profile [1,2].

This report describes a rare case of recurrent pleural solitary fibrous tumor, includes an updated review of the current literature, and, to our knowledge, is among the few published cases documenting recurrence after a prolonged disease-free interval despite initially benign histological findings.

## 2. Case Presentation

### 2.1. Medical History

**Figure 1:**
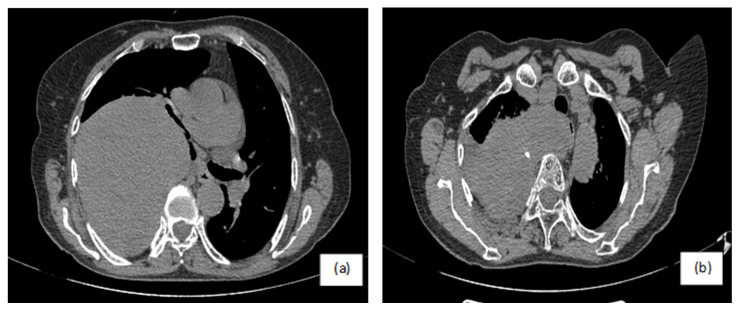
Initial chest CT from 10 years ago. (**a**) Large tumoral mass causing right main bronchus compression and mediastinal displacement toward the contralateral side; (**b**) focal calcification at the superior pole of the tumor with compressive effect on the esophagus. A small, loculated right-sided pleural effusion is also present. No evidence of mediastinal lymphadenopathy. Source: authors’ archive.

We present the case of a 78-year-old woman, a lifelong non-smoker with no occupational exposure to respiratory toxins, with a medical history notable for minimal bilateral bronchiectasis and a surgical intervention 10 years ago for a giant pleural solitary fibrous tumor measuring 19 cm × 11 cm × 11 cm and weighing 1.5 kg. The tumor was located parietally in the posterolateral hemithorax, in contact with the right upper and lower lobes, with intramediastinal extension causing contralateral displacement and compressive effects on the esophagus and right main bronchus (Figure 1).

According to the available archival pathology report, the initial tumor showed low mitotic activity (2 mitoses/10 high-power fields), absence of cytologic atypia, and no tumor necrosis. The lesion was completely excised, with no reported residual macroscopic disease; however, detailed microscopic margin assessment was not available in the archival documentation. Based on the available parameters, a retrospective Demicco score would be estimated as 5 points.

**Figure 2:**
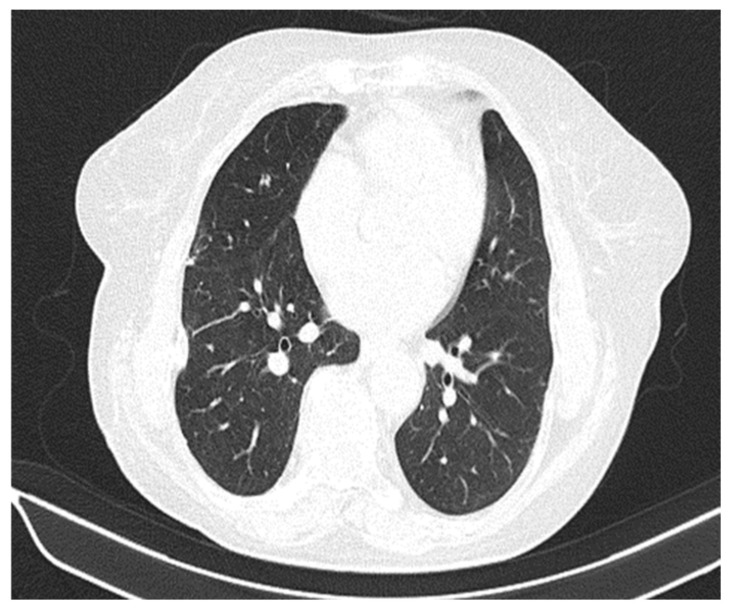
Chest CT performed one year after the first surgical intervention demonstrated a minimal pleura-based area of consolidation. Source: authors’ archive.

The patient was lost to follow-up after the initial years of imaging surveillance, during which no abnormalities were detected (Figure 2).

### 2.2. Current Clinical Presentation

A decade later, she presents to the emergency room reporting exertional dyspnea on moderate effort and right-sided pleuritic chest pain, with insidious onset and progressive worsening over the past month. On clinical examination, the only notable finding was absent breath sounds over the right hemithorax, without crackles or palpable superficial lymphadenopathy, and with vital signs within normal limits.

**Figure 3:**
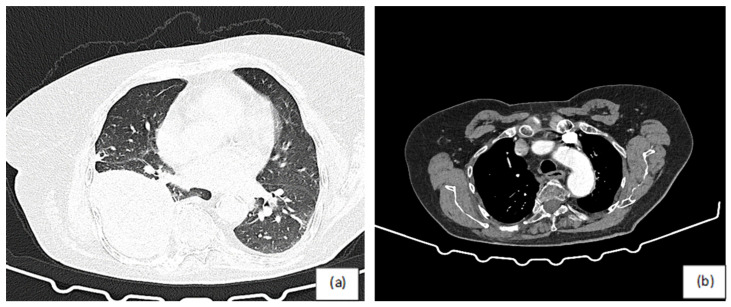
Chest CT at the current admission. (**a**) Pleura-adherent tumoral mass with smooth margins, located in the right lower lobe. (**b**) No pleural effusion or mediastinal lymphadenopathy. Source: authors’ archive.

Paraclinically, pulmonary function testing revealed a moderate obstructive ventilatory impairment, with an FEV_1_ of 70% of the predicted value. Computed tomography revealed a new round-to-oval, heterogeneous, pleura-based mass with smooth contours, measuring 12 cm × 12 cm × 10 cm, located in the right lower lobe (Figure 3).

### 2.3. Diagnostic and Treatment

Bronchoscopy of the right basal subsegmental bronchi demonstrated a slight mucosal irregularity. However, biopsy could not be performed due to marked vascularization with a mildly pulsatile character, posing a significant risk of post-procedural bleeding. Cytologic evaluation of the bronchial aspirate did not reveal malignant cells, and cultures for common bacterial flora as well as acid-fast bacilli were negative. The patient was therefore referred for a CT-guided transthoracic lung biopsy.

**Figure 4:**
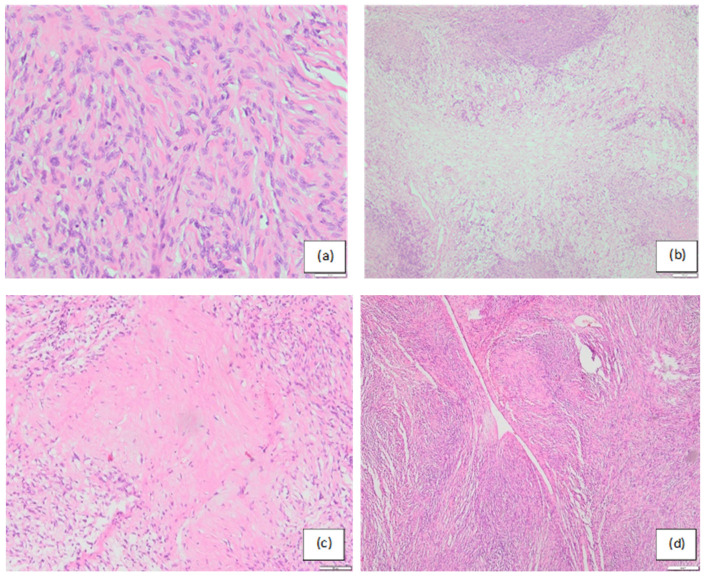
Histopathological images. Hematoxylin and eosin staining. (**a**) Spindle tumor cells and adjacent collagen fibers creating a patternless appearance—a morphological feature characteristic of SFT (×20). (**b**) Central hypocellular area with peripheral hypercellular areas (×4). (**c**) Hypocellular zones characterized by extensive collagen production (×10). (**d**) Staghorn-like vascular pattern (×10). Source: authors’ archive.

Histopathological examination (Figure 4) revealed uniform spindle cells displaying a ‘patternless’ architecture, arranged in alternating hypercellular and hypocellular areas, surrounded by keloid-type collagen and myxoid zones. Branching ‘staghorn’-type vessels were noted, with no evidence of necrosis or local pulmonary parenchymal invasion.

**Figure 5:**
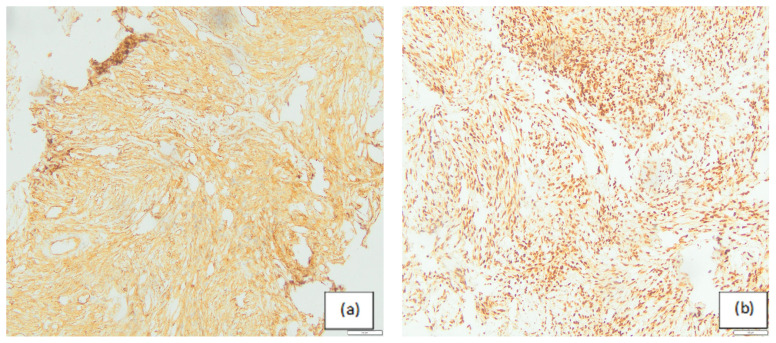
Immunohistochemical profile of the recurrent solitary fibrous tumor. (**a**) Diffuse CD34 positivity in spindle tumor cells (×10). (**b**) Nuclear STAT6 expression in tumor cells, supporting the diagnosis of solitary fibrous tumor (×10). Source: authors’ archive.

Immunohistochemistry performed on the CT-guided biopsy specimen showed diffuse CD34 positivity and nuclear STAT6 expression, supporting the diagnosis of solitary fibrous tumor (Figure 5). Additional immunohistochemical markers, including S100, GATA3, and D2-40, were negative in tumor cells, helping to exclude neural, GATA3-expressing metastatic epithelial, and mesothelial differentiation. No necrosis or mitotic activity was identified in the biopsy material. Because core biopsy specimens may not fully capture the histological heterogeneity of SFTs, definitive risk stratification was based on the postoperative resection specimen. Final microscopic evaluation confirmed recurrent solitary fibrous tumor and identified mitotic activity (4 mitoses/10 high-power fields), while necrosis and pulmonary parenchymal invasion were absent. Consequently, the final Demicco score was 5 points, corresponding to an intermediate-risk lesion (1 point for age >55 years, 2 points for tumor size between 10 and 14.9 cm, 2 points for mitotic activity, and 0 points for absence of necrosis).

**Figure 6:**
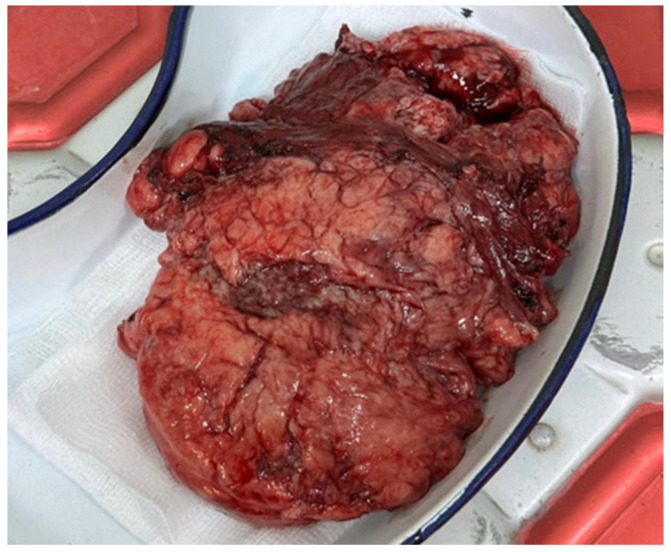
Macroscopic appearance of the tumor with an attached pedicle. Source: authors’ archive.

The patient underwent tumorectomy through a posterolateral thoracotomy, which revealed a multilobulated, 500 g mass with a pedicle arising from the parietal pleura and no evidence of adjacent pulmonary parenchymal invasion (Figure 6). The surgical margins were free of tumor infiltration, consistent with complete resection.

**Figure 7:**
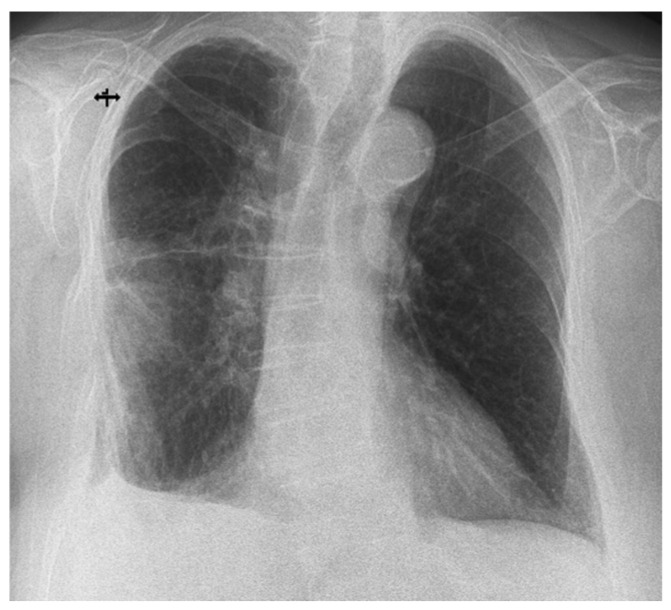
Postoperative chest radiography demonstrated satisfactory re-expansion of the right lung, with no evidence of pleural effusion. Mild residual interstitial opacities were observed in the right lower hemithorax, most likely representing postoperative sequelae and/or subsegmental atelectatic changes Source: authors’ archive.

Early postoperative imaging showed satisfactory postoperative expansion of the right lung and no relevant pleural collection (Figure 7).

She was discharged in stable hemodynamic and respiratory condition, with annual imaging follow-up advised due to several high-risk predictors for recurrence, including the tumor’s considerable size, advanced age, mitotic activity, and female sex, which has been associated in some studies with increased recurrence risk.

Thus, the present case illustrates the potential for recurrence and the unpredictable clinical course of pleural SFT, underscoring the need for long-term imaging surveillance even after ostensibly curative resections and initially favorable histological findings.

## 3. Discussion

A narrative literature review was conducted to contextualize the present case within the current evidence on pleural solitary fibrous tumors. Relevant publications were identified through searches in PubMed and major peer-reviewed medical journals using the keywords “solitary fibrous tumor”, “SFT”, “pleural fibrous tumor”, “recurrence”, “risk stratification”, and “surgery”. Priority was given to studies published between 2018 and 2025 in order to reflect contemporary classification systems, molecular insights, and management strategies.

Pleuro-pulmonary SFTs typically present with pulmonary symptoms (cough, shortness of breath, chest pain), although approximately one-third of cases are found incidentally during chest imaging [1]. The clinical presentation of solitary fibrous tumors is often nonspecific and may mimic common respiratory conditions, which can lead to delayed recognition and misdiagnosis. A differential diagnosis is essential when evaluating atypical pleura-based masses in order to distinguish SFTs from other rare neoplastic entities such as pulmonary large-cell neuroendocrine carcinoma or pulmonary amyloidoma [3,4].

According to the 2020 World Health Organization’s classification of soft tissue tumors, SFT is regarded as a fibroblastic mesenchymal tumor with intermediate biological potential; the historical term hemangiopericytoma has largely been abandoned in this context [5]. This classification reflects the fact that SFTs may behave indolently for long periods, while still retaining an unpredictable capacity for progression or late relapse. In this respect, they share conceptual similarities with other rare pulmonary mesenchymal entities with variable biological behavior, including lymphangioleiomyomatosis [6].

In the meta-analysis by Liu et al., recurrence after complete resection of pleural SFT was assessed across 23 studies, including 1262 surgically treated patients over a period spanning 1972 to 2018. Overall, recurrence occurred in 9% of cases. As expected, malignant histology was associated with a substantially higher recurrence rate than benign histology, 22% versus 3%, respectively. The anatomical origin from the parietal or visceral pleura did not significantly influence recurrence. Interestingly, female sex was also identified as a factor associated with recurrence, suggesting that biological or sex-related variables may deserve further evaluation in future risk prediction models [7]. In this context, the present case is particularly noteworthy, as it represents the recurrence of an initially benign pleural SFT—an event that is distinctly uncommon given the very low recurrence rate associated with benign histological features.

Risk stratification is preferred over anatomic staging (TNM—tumor, node, metastasis) to predict prognosis for SFTs. Importantly, the conventional histological distinction between benign and malignant SFT and the Demicco prognostic risk stratification system represent complementary, but not equivalent, classifications. A tumor may lack overt histological criteria of malignancy, such as necrosis, marked cytologic atypia, or invasive growth, while still being assigned to an intermediate-risk category according to the Demicco model, which also incorporates patient age, tumor size, and mitotic activity. Therefore, in the present case, the benign-appearing morphology and the intermediate-risk Demicco classification should not be regarded as contradictory, but rather as reflecting different aspects of tumor assessment.

The earliest widely cited approach was proposed by England et al. in 1989, and relied mainly on microscopic criteria, including cellularity, mitotic activity, pleomorphism, necrosis, and hemorrhage [8]. However, only the presence of one or more microscopic characteristics was associated with the diagnosis of high-risk (“malignant”) pleural solitary fibrous tumor. In 2002, De Perrot and colleagues introduced a four-stage pleural SFT classification that combined histological findings with macroscopic tumor morphology, distinguishing pedunculated from sessile lesions and linking these features to clinical outcomes [9]. Building on the histological criteria described by England, the authors also incorporated lesion morphology, associating pedunculated tumors with lower risk and sessile tumors with higher risk. In 2015, Tapias et al. subsequently developed a recurrence-oriented score in which six tumor characteristics were each assigned one point; a score of at least three identified patients at higher risk and supported the need for prolonged surveillance [10].

Currently, the Demicco risk stratification model published in 2017 is the most widely used in clinical practice [11]. Unlike earlier systems developed specifically for pleural tumors, this model was designed for SFTs arising at different anatomical sites, and uses age, tumor size, mitotic rate, and necrosis to classify patients as low, intermediate, or high risk. More recent work has explored whether its predictive performance can be refined by adding variables such as Ki-67 index, TERT promoter mutations, NAB2-STAT6 fusion-related data, or metabolic parameters derived from PET/CT, including SUVmax [12,13,14,15]. Table 1 summarizes the key features of the most important risk stratification models proposed over the past years.

**Figure 8:**
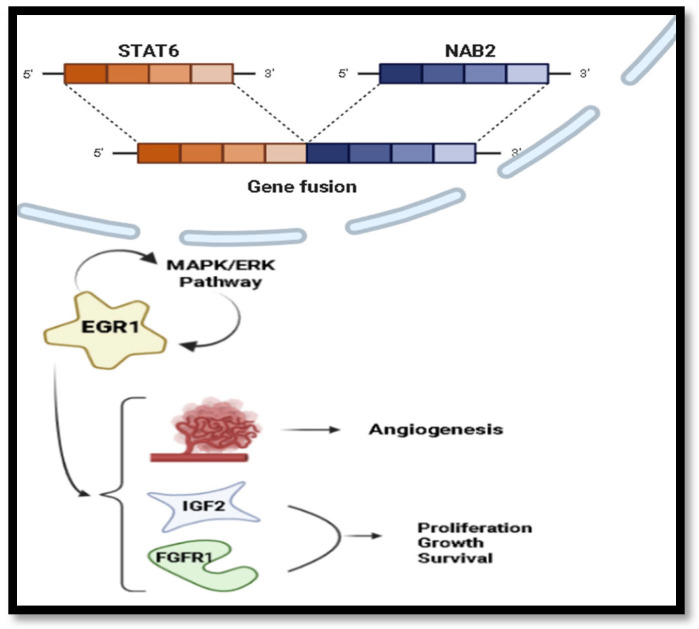
Main molecular mechanisms of SFTs via EGR1-mediated signaling and cell proliferation factors like IGF2 and FGFR1. Illustration made by authors.

From a molecular point of view, SFT is defined in most cases by a characteristic NAB2-STAT6 gene fusion generated by rearrangement on chromosome 12q. When NAB2 binds to signal transducer and activator of transcription 6 (STAT6) becomes itself an activator of transcription for early growth response 1 (EGR1), which is the key to the pathogenesis of SFTs. Once activated, EGR1 creates a positive feedback loop via the MAPK/ERK pathway, fueling tumor proliferation and angiogenesis (Figure 8) [16].

Nuclear STAT6 expression is currently one of the most reliable immunohistochemical features supporting the diagnosis of SFT, being present in the great majority of cases [16]. Additional markers frequently expressed by these tumors include CD34, BCL2, CD99, and vimentin. Conversely, markers more typical of smooth muscle, neural, or epithelial differentiation, such as actin, desmin, S100 protein, and epithelial markers, are generally absent [16].

SFT may be associated with paraneoplastic syndromes, most notably refractory hypoglycemia (Doege–Potter syndrome), which occurs in fewer than 5% of cases and is predominantly observed in large peritoneal or pleural tumors. This hypoglycemia results from tumor overproduction of high–molecular weight insulin-like growth factor-2 (IGF-2), driven by dysregulation related to the NAB2–STAT6 fusion. Surgical removal of tumor is followed by the syndrome’s resolution [17].

Another possible paraneoplastic association is secondary hypertrophic osteoarthropathy, also known as Pierre-Marie-Bamberger syndrome. It is characterized by digital clubbing, periostitis, and arthralgia or arthritis, and has been attributed to mediators produced by tumor cells, including hyaluronic acid [18,19]. Although this syndrome is classically associated with lung carcinoma, especially adenocarcinoma, it has also been described in a meaningful subset of patients with SFT [18,19].

En bloc resection with tumor-free margins represents the best predictor of good prognosis and the mainstay of treatment for SFTs, while additional hilar or mediastinal lymphadenectomy is not recommended unless there is clinical suspicion [20,21]. Intraoperative mortality in SFT resection has been reported to range between 1.5% and 12% [22]. Complete resection is required for full histopathologic evaluation. Fine-needle aspiration is often inadequately cellular, while core biopsy can establish the diagnosis but may miss features indicative of high-risk behavior due to limited sampling [1,23].

In the present case, the therapeutic approach was exclusively surgical on both occasions, consisting of tumorectomy without pulmonary resection. This strategy was supported by the absence of overt malignant features, such as necrosis or pulmonary parenchymal invasion, although the recurrent lesion fulfilled intermediate-risk criteria according to the Demicco model. Chemotherapy and radiotherapy were not considered necessary.

Video-assisted thoracic surgery, instead of thoracotomy, has been successfully performed, especially in tumors smaller than 10 cm. In the case of pedunculated tumors, an angiography to identify the pedicle, which is rich in feeding vessels, is recommended in order to perform a preoperative embolization or a thoracoscopy ligation of vessels. In sessile SFT, embolization is the most effective procedure in reducing intraoperative bleeding [24,25,26].

For patients with resected SFT and certain higher-risk features (e.g., positive surgical margins, high mitotic count), the use of adjuvant radiotherapy may prevent local recurrences, although an overall survival benefit has not been established in observational studies. Due to the limited prospective evidence, its use should be discussed within a multidisciplinary team, and individualized for each patient [27].

Similar to radiotherapy, the role of adjuvant chemotherapy in advanced, inoperable, and/or metastatic disease has not been fully clarified and is not standardized. The most common regimens that have been reported are dacarbazine as a single agent or in combination with doxorubicin [28]. More recently, research has been focused on the possible use of antiangiogenic therapy for patients with progressive disease on chemotherapy, such as sunitinib, pazopanib, and temozolomide in combination with bevacizumab [28]. For patients previously treated with dacarbazine (with or without doxorubicin), it is preferred to pazopanib, which is now in phase 2 trial [29]. Despite promising results, the data is currently not sufficient to establish new standards of care.

## 4. Limitations

The main limitation of this report is its single-case design, which prevents generalization regarding recurrence risk. Although the manuscript discusses relevant molecular pathways, no extended molecular profiling beyond STAT6 immunohistochemistry—such as TERT promoter mutation analysis or detailed proliferative index assessment—was performed, which may have provided additional prognostic information. Furthermore, long-term follow-up after the second surgical intervention is currently ongoing, and continued surveillance will be necessary to more comprehensively evaluate the patient’s long-term outcome.

## 5. Conclusions

This case documents the late recurrence of a benign-appearing pleural SFT and emphasizes the need to integrate morphology, immunohistochemistry, surgical pathology, and prognostic risk models. The recurrent lesion lacked necrosis and invasive growth, but mitotic activity in the resected specimen contributed to an intermediate-risk Demicco classification. Complete surgical resection remains the cornerstone of management and provides the most reliable prognostic information. However, as illustrated by this single case, recurrence may occur after a prolonged disease-free interval, supporting the consideration of extended imaging surveillance, particularly in patients with large tumors or other adverse clinicopathological features. A multidisciplinary approach remains essential to ensure optimal diagnostic assessment, therapeutic decision-making, and long-term patient management.

## Figures and Tables

**Table 1:** Main risk stratification models for solitary fibrous tumor.

Model (Author, Year)	Target Population	Key Variables	Risk Categories/Stages	Cut-Off/Definition
England et al., 1989 [8]	Pleural SFT	Mitoses > 4/10 HPF	Benign VS Malignant	Malignant if ≥1 adverse microscopic feature
		Necrosis Hypercellularity		
		Nuclear pleomorphism Hemorrhage		
de Perrot et al., 2002 [9]	Pleural SFT	Mitoses > 4/10 HPF Necrosis Hypercellularity	Stage I–IV	I: pedunculated benign II: sessile benign III: pedunculated malignant IV: sessile malignant
		Nuclear pleomorphism Resection margins Stromal/vascular invasion		
Tapias et al., 2015 [10]	Pleural SFT	Mitoses > 4/10 HPF Necrosis Hypercellularity/ Hemorrhage Pleural origin Pedunculated/sessile Tumor size	Low risk (0–2) High risk (≥3)	1 point per feature; ≥3 = high-risk recurrence
Demicco et al., 2017 [11]	Extrameningeal SFT (any site)	Mitoses Necrosis Age Tumor size	Low risk (0–3) Intermediate risk (4–5) High risk (6–8)	Age ≤55 = 0; >55 = 1. Size <5 = 0; 5–9.9 = 1; 10–14.9 = 2; ≥15 = 3. Mitoses 0–3 = 0; 4–9 = 1; ≥10 = 2. Necrosis <10% = 0; ≥10% = 1.

## Data Availability

All data generated or analyzed during this study are included in this published article.

## Abbreviations

The following abbreviations are used in this manuscript:

NAB2	Binding protein 2
STAT6	Signal transducer and activator of transcription
TERT	Telomerase reverse transcriptase
SFT	Solitary fibrous tumor
VS	Versus
